# Supplementary material for: Toward High-Voltage Cathodes for Zinc-Ion Batteries: Discovery Pipeline and Material Design Rules
Source: Chem Mater. 2025 Aug 13;37(16):6213–26. doi: 10.1021/acs.chemmater.5c00916 (PMC12392462; doi:10.1021/acs.chemmater.5c00916)
Supplement: Supplementary file 1 [file cm5c00916_si_001.pdf]

Supporting Information: Toward High-Voltage Cathodes for Zinc-Ion Batteries: Discovery Pipeline and Material Design Rules

Roberta Pascazio,[†,‡,¶] Qian Chen [§],[†,‡,¶] Haoming Howard Li,[†,‡] Aaron D. Kaplan,[‡] and Kristin A. Persson[*,†,‡]

†*Department of Material Science and Engineering, University of California, Berkeley, CA 94720, U.S.A.*

‡*Materials Science Division, Lawrence Berkeley National Laboratory, Berkeley, 94720, United States*

¶*R.P. and Q.C. contributed equally to this work.*

E-mail: kristinpersson@berkeley.edu

S1 Screening criteria

The following sections detail the criteria applied for all tiers in the screening protocol.

S1.1 Tier 1: Property Screening

Composition screening. The composition screening evaluated the following criteria: (i) the presence of at least one transition metal capable of undergoing reduction reactions upon Zn insertion (Ti, V, Cr, Mn, Fe, Co, Ni, Cu, Nb, Mo, Ru, Ag, W, Re, Sb, and Bi); (ii)

the presence of either O or S in the host structure; (iii) the absence of radioactive, toxic, and/or expensive elements, including As, Au, Ir, Pt, Pd, Rh, Tc, and elements with atomic number greater than 83. The exclusion criteria and toxicity hazards are in agreement with publicly available safety data on elements (e.g., NIOSH IDLH thresholds[S1]). However, we caution that oxidation state-specific effects were not considered in the toxicity tier due to their ambiguity in high-throughput DFT screening as well as uncertainty of synthesis and recycling conditions; (iv) the absence of Zn or other intercalating ions, such as H, Li, Na, Ca, Mg, and K, to simplify the evaluation of ion mobility and diffusivity. This screening narrowed the pool of potential candidates from 163,109 to 22,769 structures.

Stability/synthesizability screening. In this tier, materials were screened for (i) an energy above the hull[a] < 0.1 eV/atom, in agreement with established thresholds:[S3,S4] structures that are unstable in their charged state are unlikely to be stable during the discharge process; (ii) aqueous instability < 0.5 eV/atom, measured at 1.5 V vs. Zn/Zn^{2+} and a pH range of 5-5.5, ensuring that the material does not decompose in water and that stable solid phases are present in decomposed products. The `PourbaixDiagram` module in `pymatgen`[S5,S6] is employed to calculate the decomposition energy, stable products and decomposition voltage of the materials, infering their aqueous stability in the outlined conditions. The choice of looser criteria on aqueous stability is motivated by the possible formation of passivation layers on the materials, which would allow for their stability in aqueous media.[S7] This screening reduced the number of candidates from 22,769 to 6,980 viable materials.

Practical screening. This stage screened the following properties: (i) a price per unit capacity $<$ \$0.005/mAh, excluding systems with a large fraction of expensive elements from the database; (ii) gravimetric capacity > 100 mAh/g, motivated by the search for high-performance materials. This final screening tier reduced the optimal candidates from 6,980 to 4,297 structures. Two further screening criteria were applied to the selected structures: a (iii) structural screening, in which only one representative structure was selected for materi-

[a]The energy above the hull is defined as the distance of a phase from the hull composed by its most stable phases, and it is a measure of its thermodynamic stability.[S2]

als with the same formula/structural framework but different transition metal (TM) ordering or ratios (e.g., NASICON structures $(TM)_2(PO_4)_3$ with different TM combinations). This screening was performed using structure matching capabilities in `pymatgen`;[S5] a (iv) TM screening, in which systems containing Mn, Co, Cr, and Ni were prioritized due to their favorable voltages and high material availability.

The 4,297 structures were thus reduced to 1,181 distinct crystal structure types.

Overall, this final screening tier reduced the optimal candidates from 6,980 to 1,181 structures with distinct crystal structures.

S1.2 Tier 2: ion insertion

S1.2.1 Additional Screening and Prototype Matches

The 313 intercalated materials presenting successful insertion electrode calculations were further screened based on the same stability and synthesizability criteria employed in **Tier 1** of the screening, with the addition of the following criteria: (i) an energy above the hull < 0.1 eV/atom for all the structures obtained in the insertion algorithm to construct voltage pairs; (ii) average intercalation voltage > 1.3 V vs. Zn/Zn^{2+}; (iii) gravimetric capacity > 160 mAh/g; (iv) energy density > 300 Wh/kg. These criteria were chosen to target high voltage and energy density cathodes. Finally, we required (v) a volume change upon intercalation lower than 20% to account for the typically higher volume changes seen in Zn^{2+} intercalation compared to the 5-10% common in LIBs.[S4,S8–S10] In addition to these requirements, the best candidates were tested for their conversion voltage using data from the Materials Project phase diagram[S2,S11] and `pymatgen`.[S5] The cutoff chosen for the conversion voltage was such that the difference between the conversion and intercalation voltages could not exceed 0.5 V. The threshold of 0.5 V is based on DFT-calculated data which has been benchmarked on experimental values.[S12]

These conditions, which are summarized in Figures S1-S6, ensured that the candidate cathodes fit within high performance and stability standards, as well as within the thermo-

dynamic stability window of common aqueous electrolytes.[S7,S13] More, they introduce new screening criteria which had not been considered in previous multivalent cathode pipelines.[S14,S15] This screening Tier reduced the 226 candidates obtained in Tier 2 to the 37 best performing materials. In particular, of the 37 materials, 33 (89%) met the conversion voltage criterion. This result represents a significant improvement over Tier 2 candidates (for which only 51%, corresponding to 116 materials had passed this threshold), underscoring the effectiveness of using thermodynamic criteria to identify promising materials.

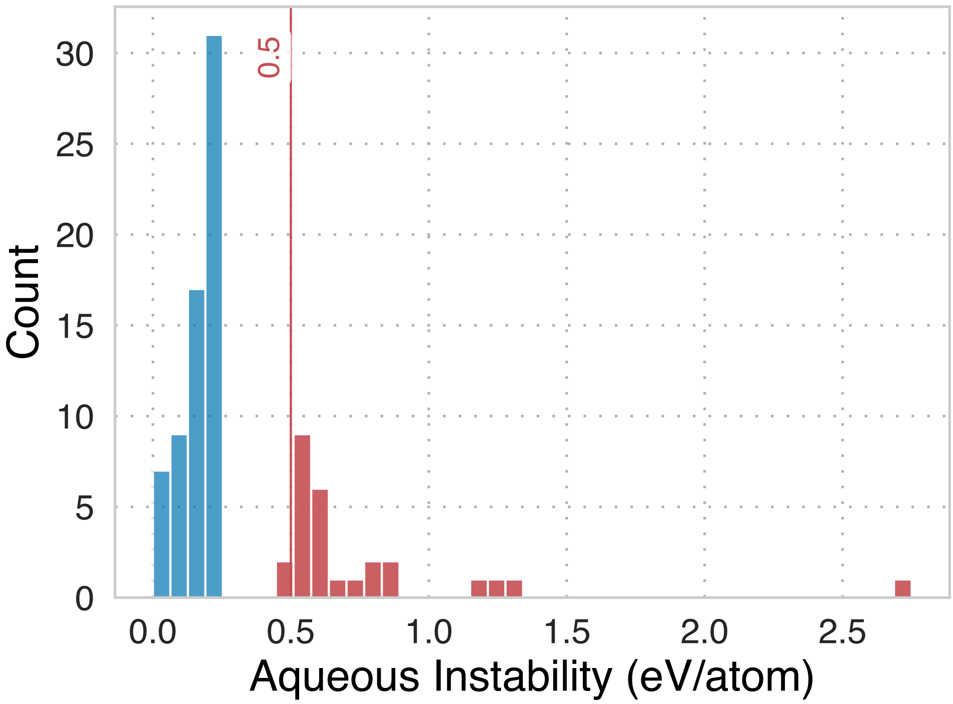

Figure S1: Maximum aqueous instability among all structures obtained from the insertion algorithm in each framework. Threshold = 0.5 eV/atom.

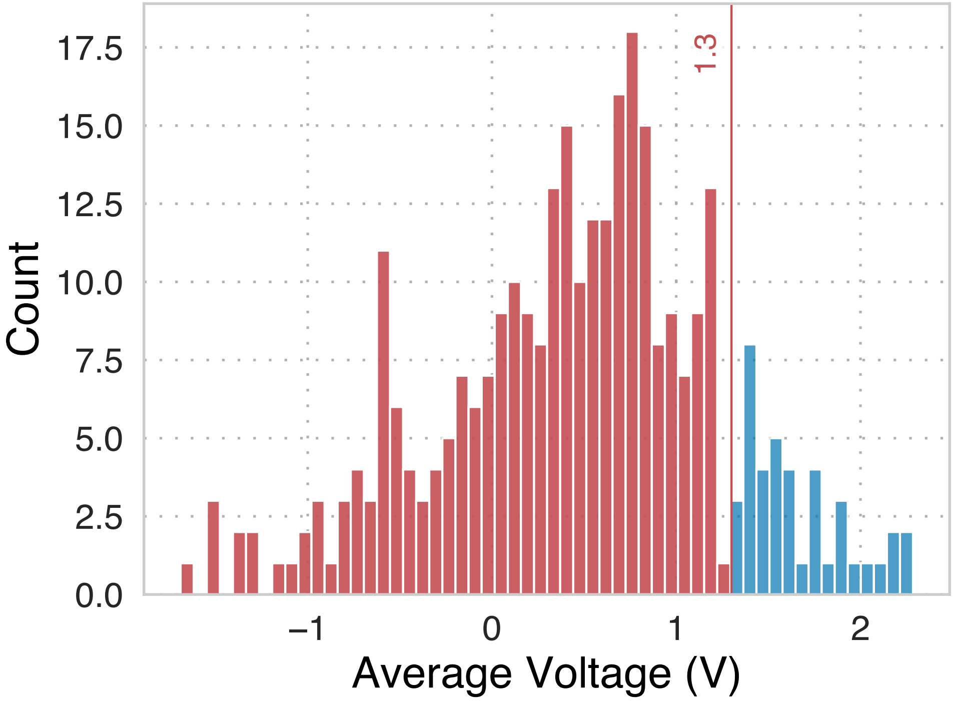

Figure S2: Average intercalation voltage distribution. Threshold = 1.3 V.

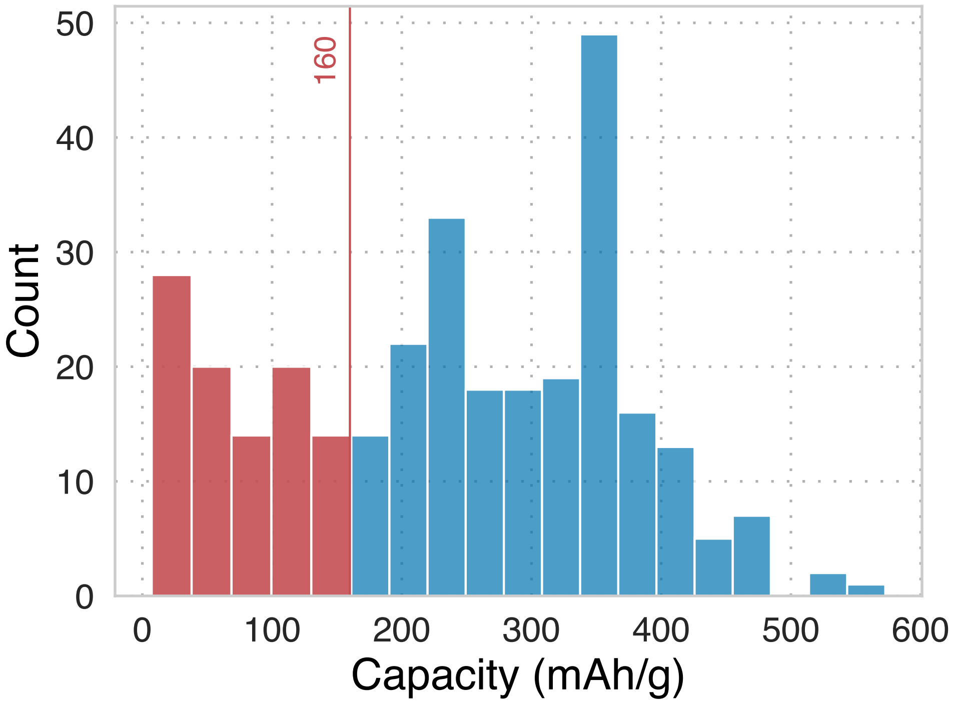

Figure S3: Gravimetric capacity distribution. Threshold = 160 mAh/g.

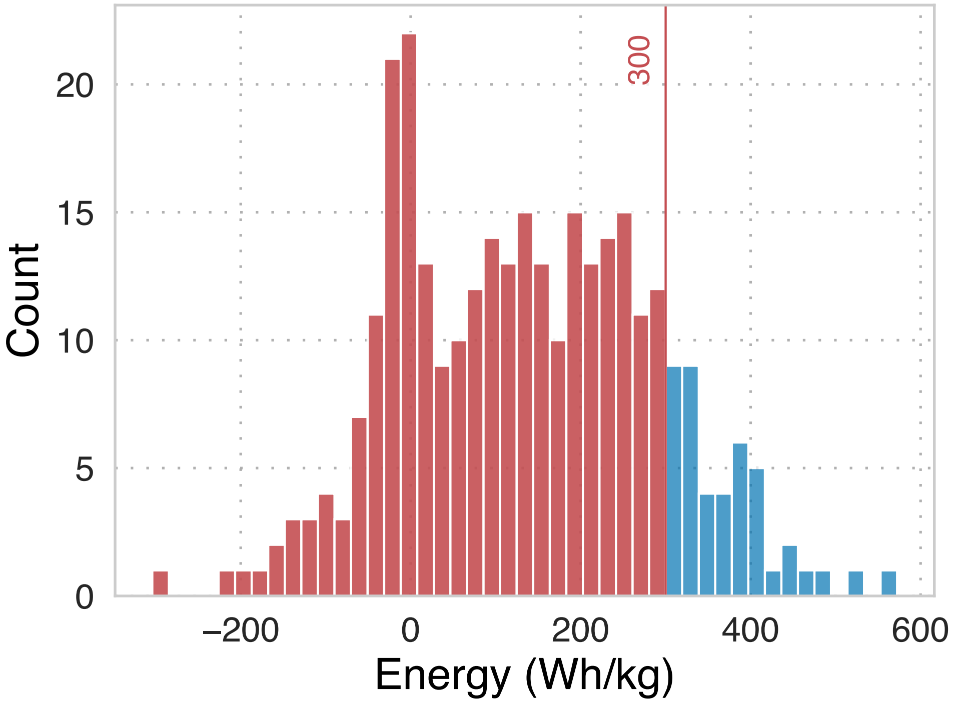

Figure S4: Gravimetric energy density distribution. Threshold = 300 Wh/kg.

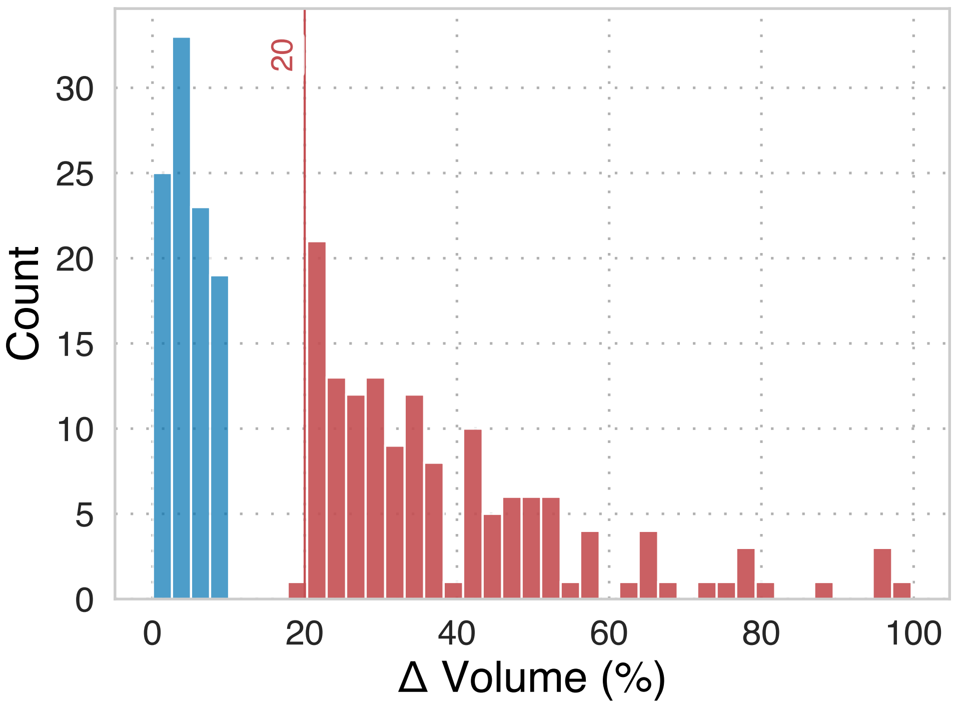

Figure S5: Distribution of maximum volume change (ΔV, %) observed during cycling. Threshold = 20%.

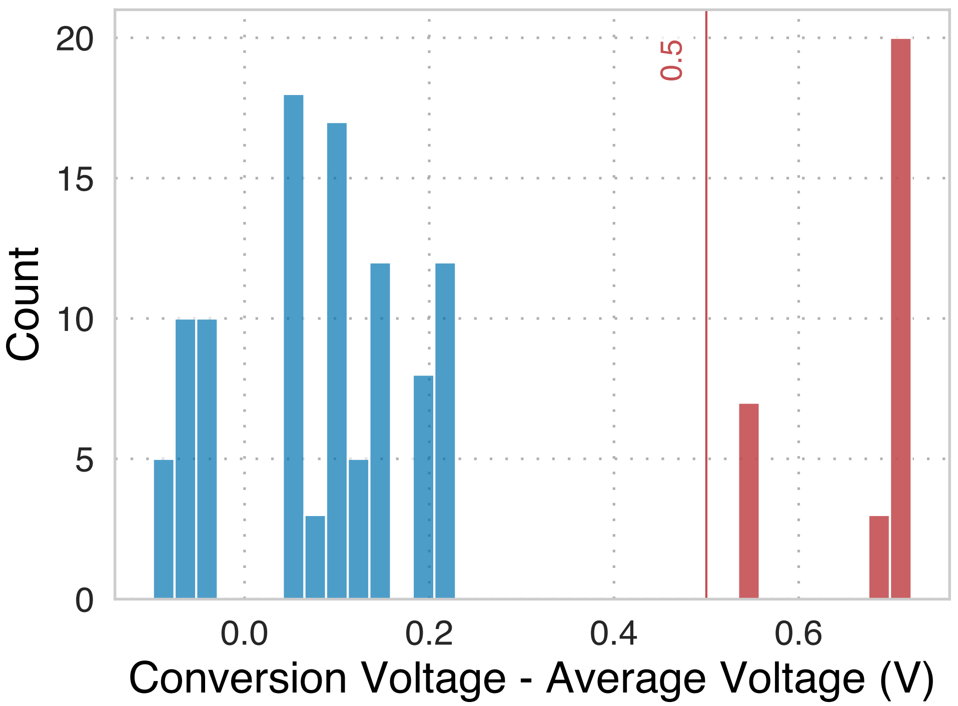

Figure S6: Distribution of difference between conversion and intercalation voltages (on best candidates resulted by the additional screening/refinement tiers). Threshold = 0.5 V.

S2 Literature Background

The following section provides literature background on the state-of-the-art research on the most promising candidate materials and their ICSD matches in batteries involving Zn and other working ions.

As detailed in Section **Screening Results**, the top four candidate materials belong to well-established cathode frameworks for multivalent ion batteries, such as NASICONs and tavorites.[S16]

$CoPO_4F$ (**mp-25444**) and $CrOPO_4$ (**mp-772173**) both structurally belong to the tavorite family (general formula $M(XO_4)Y$, where M = Fe, V, Ti, Mn, Co, ..., X = P, S, W, ..., and Y = F, O, OH[S16]) Tavorite frameworks have been extensively studied for their high structural tolerance to intercalation and open channels for ion diffusion, enhancing intercalation kinetics and potentially enabling multidirectional transport.[S17]

Notable examples for Y = F which are chemically and structurally similar to $CoPO_4F$ include VPO_4F and $FePO_4F$, which have shown promise as cathodes in sodium-ion batteries.[S18,S19]

For instance, Na_2FePO_4F exhibits a notable experimental discharge capacity of 117 mAh/g, good capacity retention, and a small volume change during the charge/discharge process (∼4%),[S19] while vanadium-based polyanion compounds of general formula $AVPO_4F$ (A = Li, K) have been synthesized using a microwave-assisted solvothermal approach and explored for their electrochemical behavior in Li and Na-ion batteries, maintaining 75% of the initial specific capacity (111 mAh/g) up to 100 cycles.[S18] Building on the success of VPO_4F and $FePO_4F$, several orthorhombic polymorphs of $CoPO_4F$ (*Pnma*, *Pbcn*) have been synthesized through solid-state reactions and investigated as high-voltage cathodes for monovalent ions.[S20–S25] The *Pnma* phase, in particular, has undergone extensive chemical and thermal stability testing and has a theoretical capacity of 287 mAh/g for two Li^+ ions at potentials between 4.2–5.1 V vs. Li/Li^+, achieving an experimental capacity of 140 mAh/g for one Li atom by mitigating the influence of side reactions.[S23,S24] Slightly lower theoretical (122

mAh/g per Na atom, or 244 mAh/g for 2 Na) and experimental capacities (213 mAh/g at an average potential of 4.3 V vs. Na/Na^{+}) were reported for the sodiated version of this polymorph, Na_2CoPO_4F.[S25] However, these capacities are experimentally limited by the de/intercalation of the second working ion atoms, which fall outside the voltage stability range of most commercial electrolytes. Improvements have been seen with *in-situ* carbon modification, achieving 107 mAh/g per Na atom[S26] in Na_2CoPO_4F by coating the particles and employing a high-voltage electrolyte.

In a parallel fashion to what is reported for $CoPO_4F$, for $CrOPO_4$, similar structures of the general formula MOPO$_4$ - most notably $VOPO_4$ polymorphs - have recently gained prominence as cathode materials for both monovalent[S27–S29] and divalent[S30,S31] ion batteries, showing particular promise as Mg cathodes (for which theoretical capacities of 288 mAh/g have been calculated at an average voltage of 2.7 V vs. Mg/Mg^{2+}[S30]). These successes have inspired further exploration of substituted $V_yM_{1-y}OPO_4$ compounds, such as $LiV_yCr_{1-y}OPO_4$, with promising results: good capacity retention (92% after 50 cycles), although parasitic side reactions linked to structural strain have been observed in the system.[S32]

While the triclinic polymorphs of $CoPO_4F$ and $CrOPO_4$ have both been previously explored computationally in high-throughput searches for Li-ion battery cathodes ($CoPO_4F$ showed a 7% volume change and 589 Wh/kg energy density, and was predicted as likely synthesizable - i.e. predicted to release less than 30 meV per atom upon decomposition. $CrOPO_4$, on the other hand, showed a 6% volume change),[S33] $CoPO_4F$ (**mp-25444**) and $CrOPO_4$ (**mp-772173**) have yet to be synthesized or tested for electrochemical performance.

However, their frameworks match ICSD entries such as $SbOPO_4$ (**icsd-201743**, **mp-9750**), a high-temperature polymorph of $NbOPO_4$, β-$NbOPO_4$ (**icsd-93766**, **icsd-40870**, **icsd-93767**, **icsd-252566**, **mp-542453**), and the α-polymorph of $VOPO_4$, all of which have been previously investigated as intercalation electrodes.[S19,S30,S34] As stated previously, $VOPO_4$ polymorphs (α-, β-, and ϵ-$VOPO_4$ in particular) have been widely investigated as

cathodes for Li- and Na-ion batteries.[S27–S29] For divalent ions, ϵ-$VOPO_4$ shows promise as a Mg cathode, with a theoretical capacity of 288 mAh/g at an average voltage of 2.7 V vs. Mg/Mg^{2+}.[S30] Similarly, α- and δ-$VOPO_4$ have been proposed as Ca cathodes, with δ-$VOPO_4$ also being tested for aqueous Zn-ion batteries, achieving a capacity retention of 91 mAh/g after 1000 cycles at an average voltage of 1.46 V vs. Zn/Zn^{2+}.[S31]

Unlike its monoclinic counterpart, the orthorhombic $CrOPO_4$ polymorph (**mp-26924**) does not strictly belong to the tavorite family, as this form features a more distorted phosphate framework deviating from the classic tavorite arrangement.

However, an ICSD match aligns this structure with the symmetry group of the β-polymorph of $VOPO_4$, (**icsd-291605**, **icsd-9413**, **mp-25265**), whose properties have been discussed previously. β-$VOPO_4$ is a known framework for 3D ion migration, showing preferential ion diffusion along the b-axis,[S19,S30,S35] and a widely-employed cathode for multivalent ions (Mg, Ca), as well as monovalent ions like Li and Na,[S27–S29] showing capacities of 118.6 mAh/g at average 4 V vs. Li/Li^{+}[S36] and making it reasonable to anticipate a similar behavior from the $CrOPO_4$ polymorph.

Lastly, $Mn_2(PO_4)_3$ (**mp-26062**) structurally belongs to NASICON materials (general formula $M_2(XO_4)_3$, where M = Fe, V, Ti, Mn, Co, ... X = P, S, W, ...[S16]). NASICON materials are known electrode materials, and various synthetic strategies (carbon coating, doping) and theoretical guidelines[S37,S38] have been developed to enhance their electrochemical performance,[S39,S40] their structural stability and reversibility during cycling, particularly in applications for Li and Na-ion batteries. While specific diffusion pathways for $Mn_2(PO_4)_3$ have yet to be reported, NASICON-type phosphates with characteristic rhombohedral symmetry (e.g. $Fe_2(PO_4)_3$, $Ti_2(PO_4)_3$) have been widely investigated as cathode materials for both Li and Na ion batteries. For instance, $Li_3Fe_2(PO_4)_3$ demonstrates a theoretical capacity of 128 mAh/g at an average voltage of 2.85 V vs. Li/Li^{+},[S16,S41] while $NaTi_2(PO_4)_3$

has a theoretical capacity of 133 mAh/g at an average potential of 2.1 V vs. Na/Na^+.[S42] Both materials show excellent reversibility over repeated cycling.[S43,S44] In addition to these well-studied systems, a number of NASICON-type phosphates incorporating Mn and other transition metals have also been explored: a theoretical capacity of 117 mAh/g at 2.1 V vs. Na/Na^+ has been reported for $Na_3MnTi(PO_4)_3$ for the Mn-based redox couples,[S45] and a satisfactory theoretical capacity of 111 mAh/g (attributed to the reversible intercalation/deintercalation of two Na-ions), with a high redox potential of 3.6 V vs. Na/Na^+.[S46,S47] Good performances were also reported for high-entropy NASICON phosphates of mixed compositions.[S48]

$Mn_2(PO_4)_3$ matches to an ICSD prototype, $Nb_2(PO_4)_3$ (**icsd-65658**, **mp-17242**), a known anode material in Li- and Na-ion batteries[S49] characterized by high capacities (227 mAh/g at average voltage of 1.86 V vs. Li/Li^+ for Li, 179 mAh/g at at average voltage of 1.46 V vs. Na/Na^+ for Na for the first charge/discharge cycle, with good capacity retention, $\sim$ 60.2% after 200 cycles) and diagonal diffusion paths, suggesting that a similar diffusion behavior might be expected for $Mn_2(PO_4)_3$.

S3 Zn^{2+} Migration: Pathways and Hops

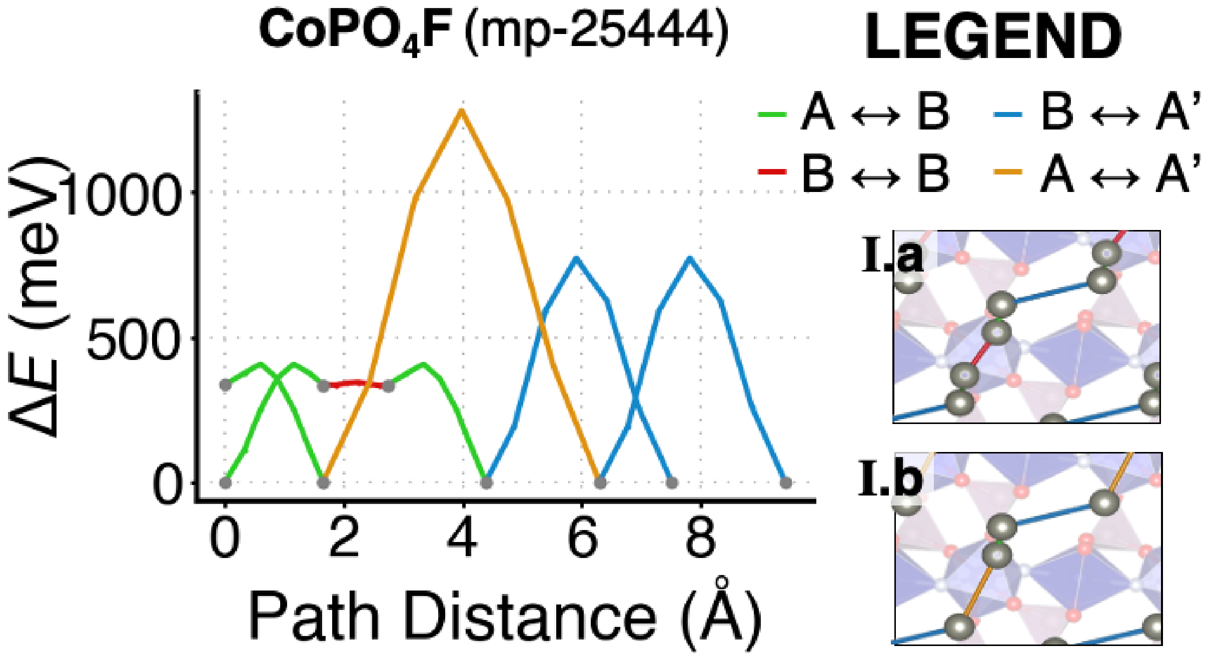

Figure S7: Energy landscape plots for triclinic tavorite $CoPO_4F$ (mp-25444), exhibiting energetic barriers of 772 meV and 1283 meV over total path distances of respectively 7.51 Å and 9.43 Å (both **PathwayI**, left and right).

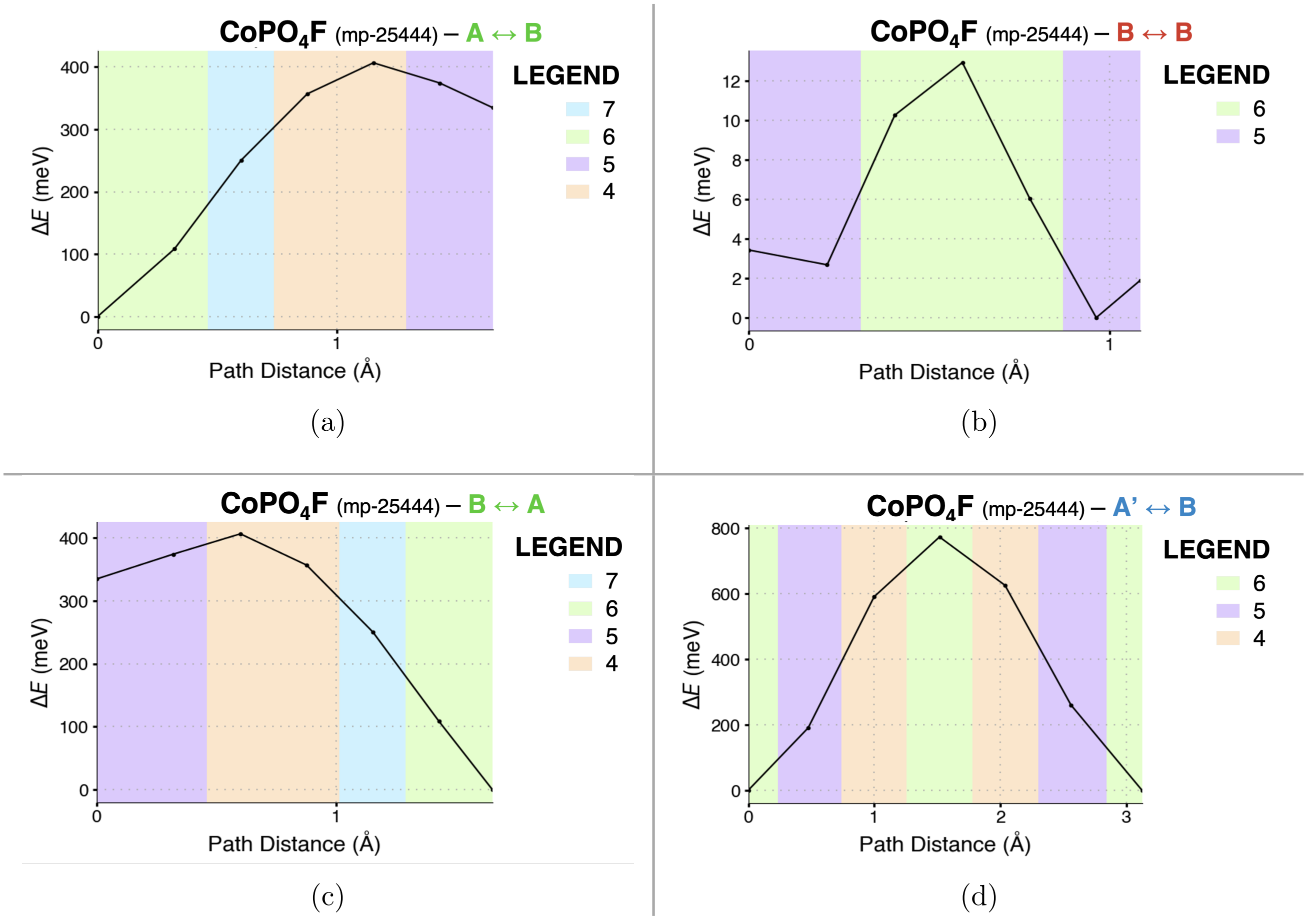

Figure S8: Energy landscape plots for Zn^{2+} migration along **PathwayI.a** in $CoPO_4F$ (mp-25444).

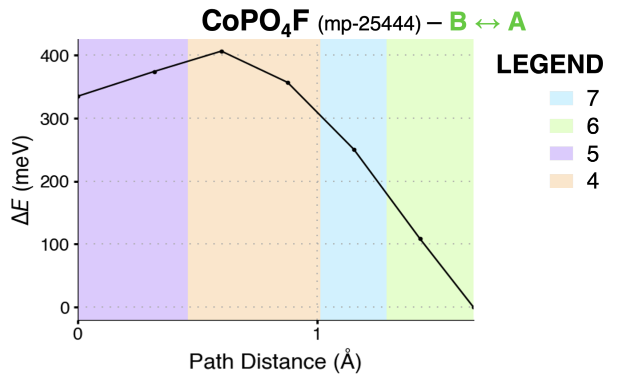

(a)

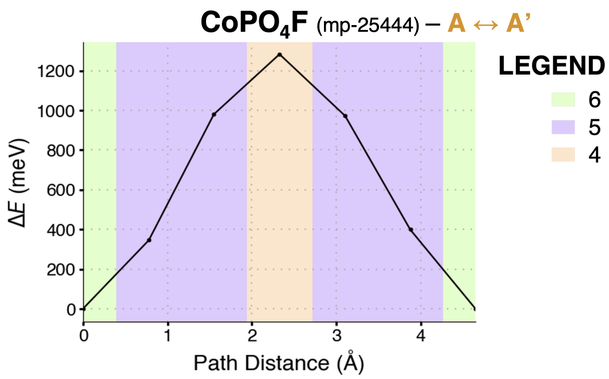

(b)

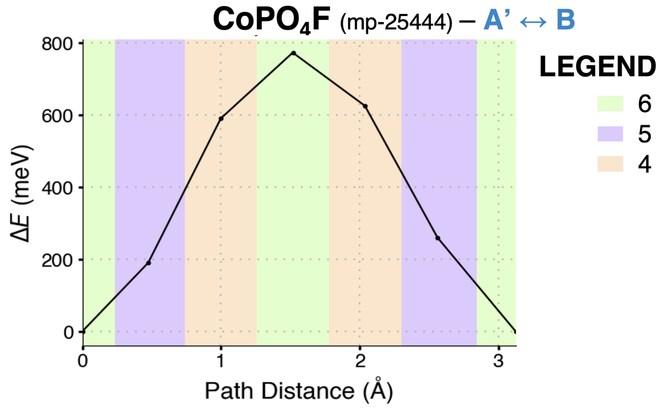

(c)

Figure S9: Energy landscape plots for Zn^{2+} migration along **PathwayI.b** in $CoPO_4F$ (mp-25444).

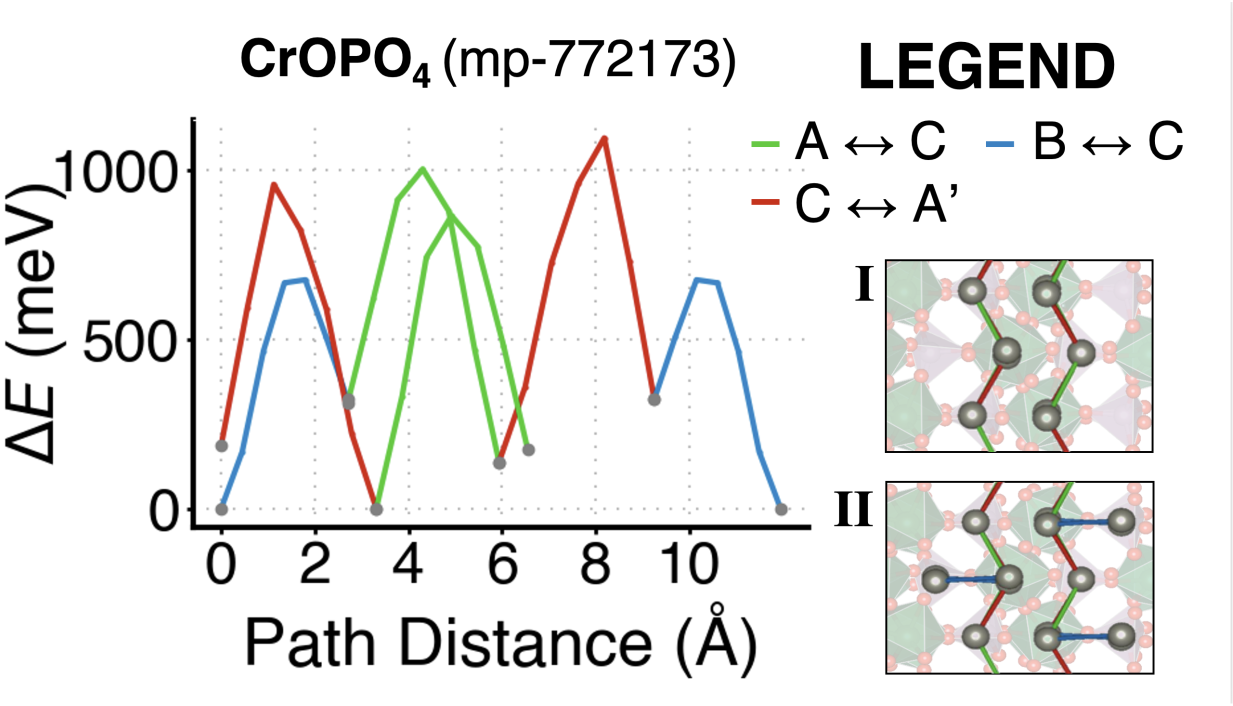

Figure S10: Energy landscape plots for monoclinic tavorite $CrOPO_4$ (mp-772173), both with an energetic barrier of 958 meV, across total path distances of respectively 6.55 Å (**PathwayI**, on the left) and 11.95 Å (**PathwayII**, on the right).

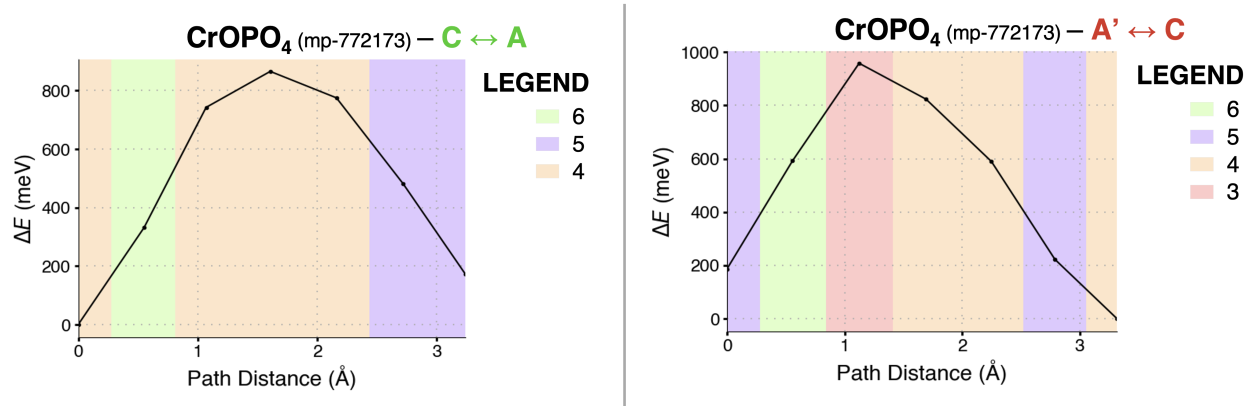

(a) (b)

Figure S11: Energy landscape plots for Zn^{2+} migration along **PathwayI** in $CrOPO_4$ (mp-772173).

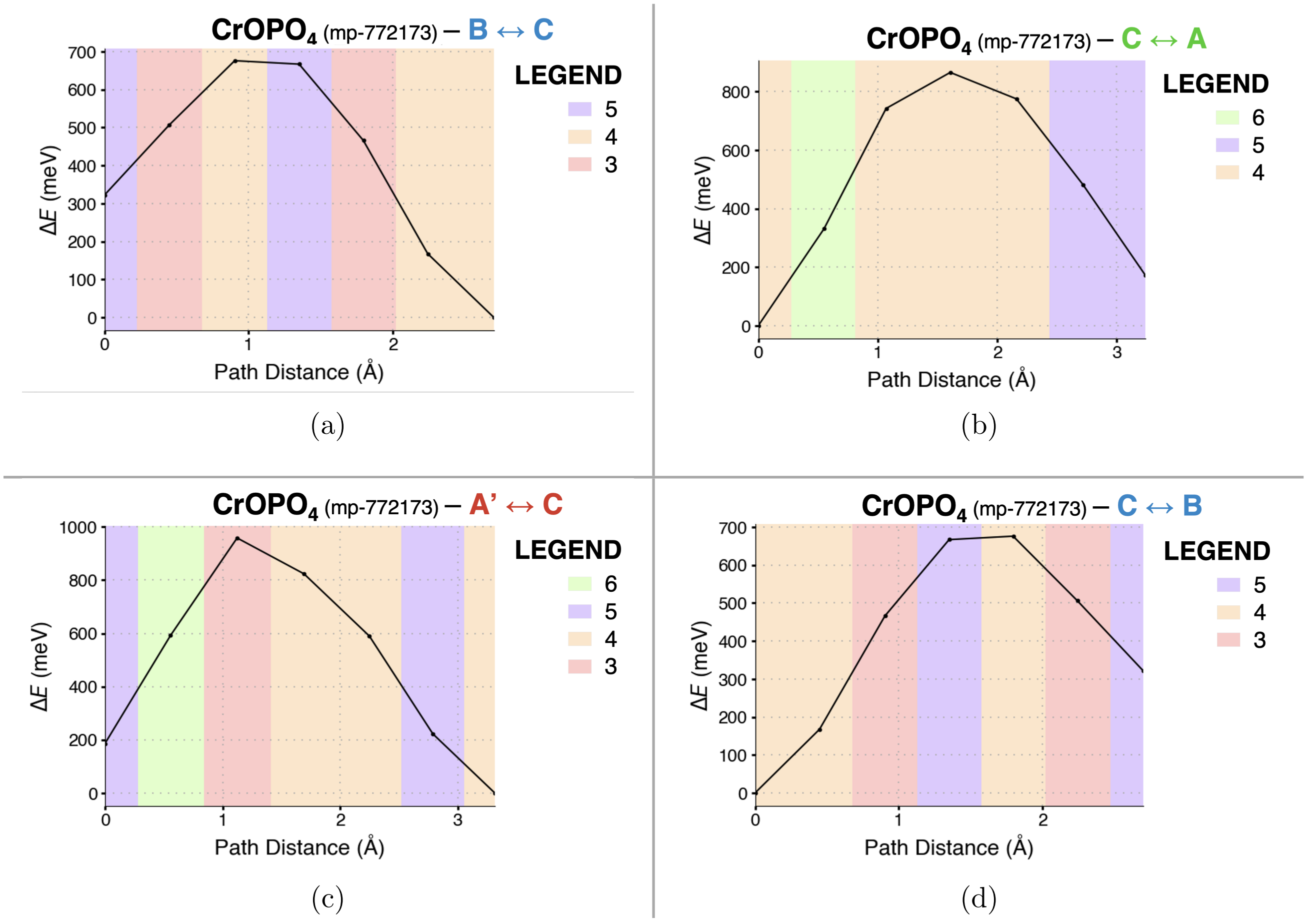

Figure S12: Energy landscape plots for Zn^{2+} migration along **PathwayII** in $CrOPO_4$ (mp-772173).

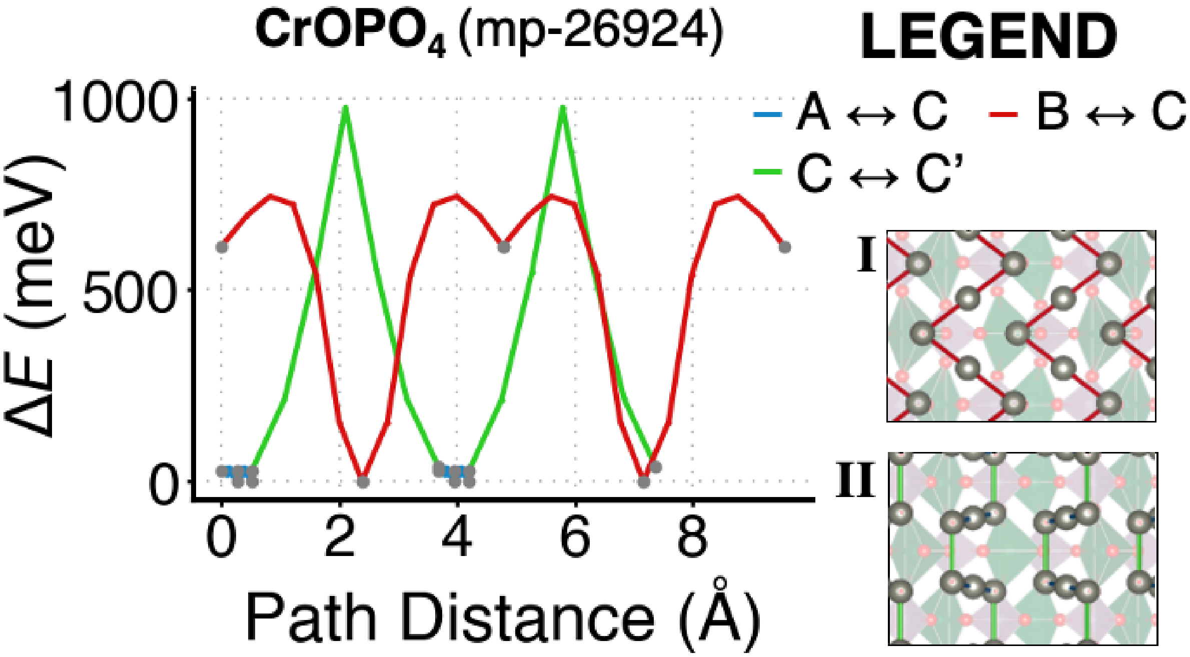

Figure S13: Energy landscape plots for orthorhombic Cr phosphate $CrOPO_4$ (mp-26924), showing energetic barriers of 774 meV and 951 eV along a total path distance of respectively 9.55 Å (**PathwayI**, on the left) and 7.39 Å (**PathwayII**, on the right).

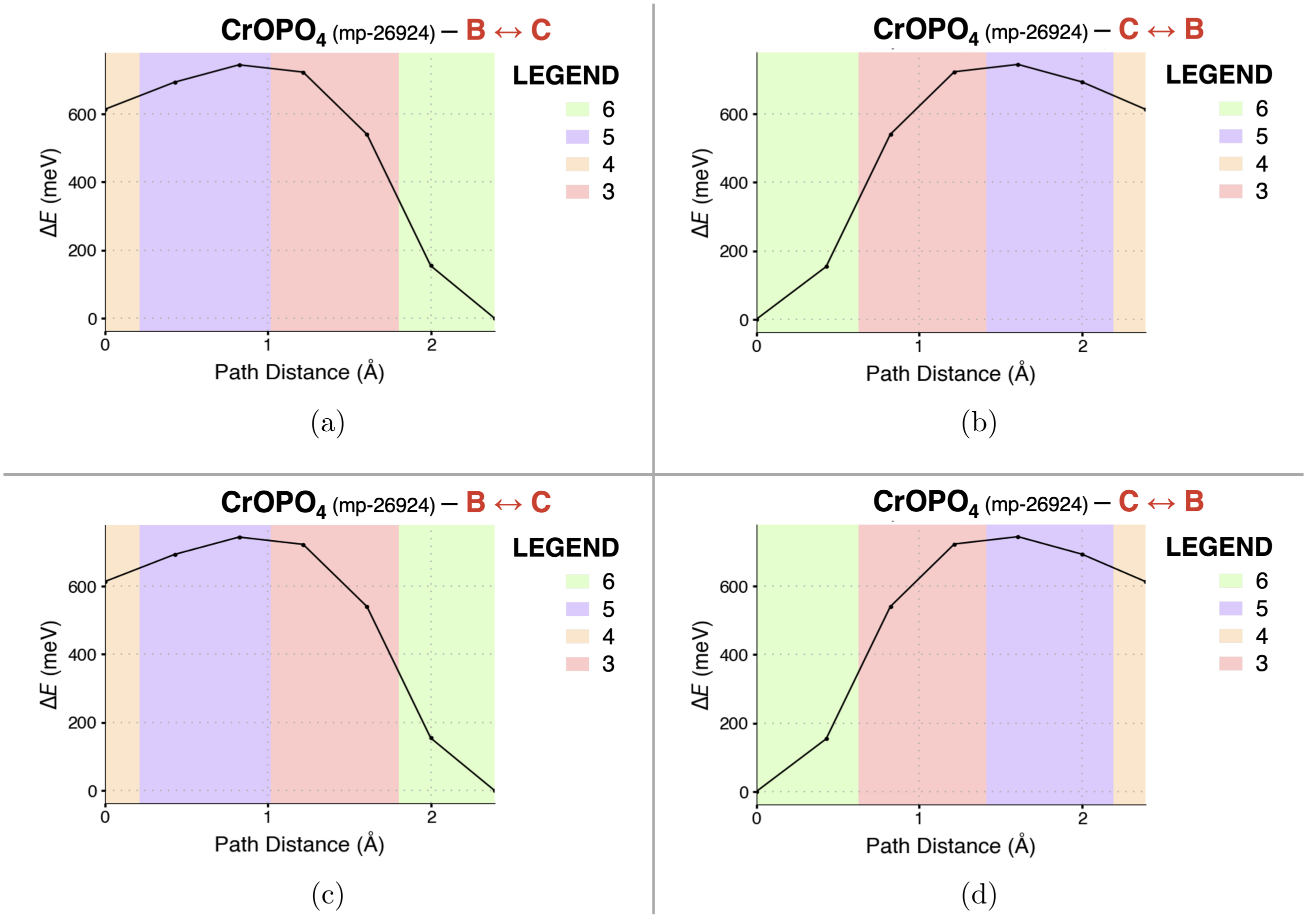

Figure S14: Energy landscape plots for Zn^{2+} migration along **PathwayI** in $CrOPO_4$ (mp-26924).

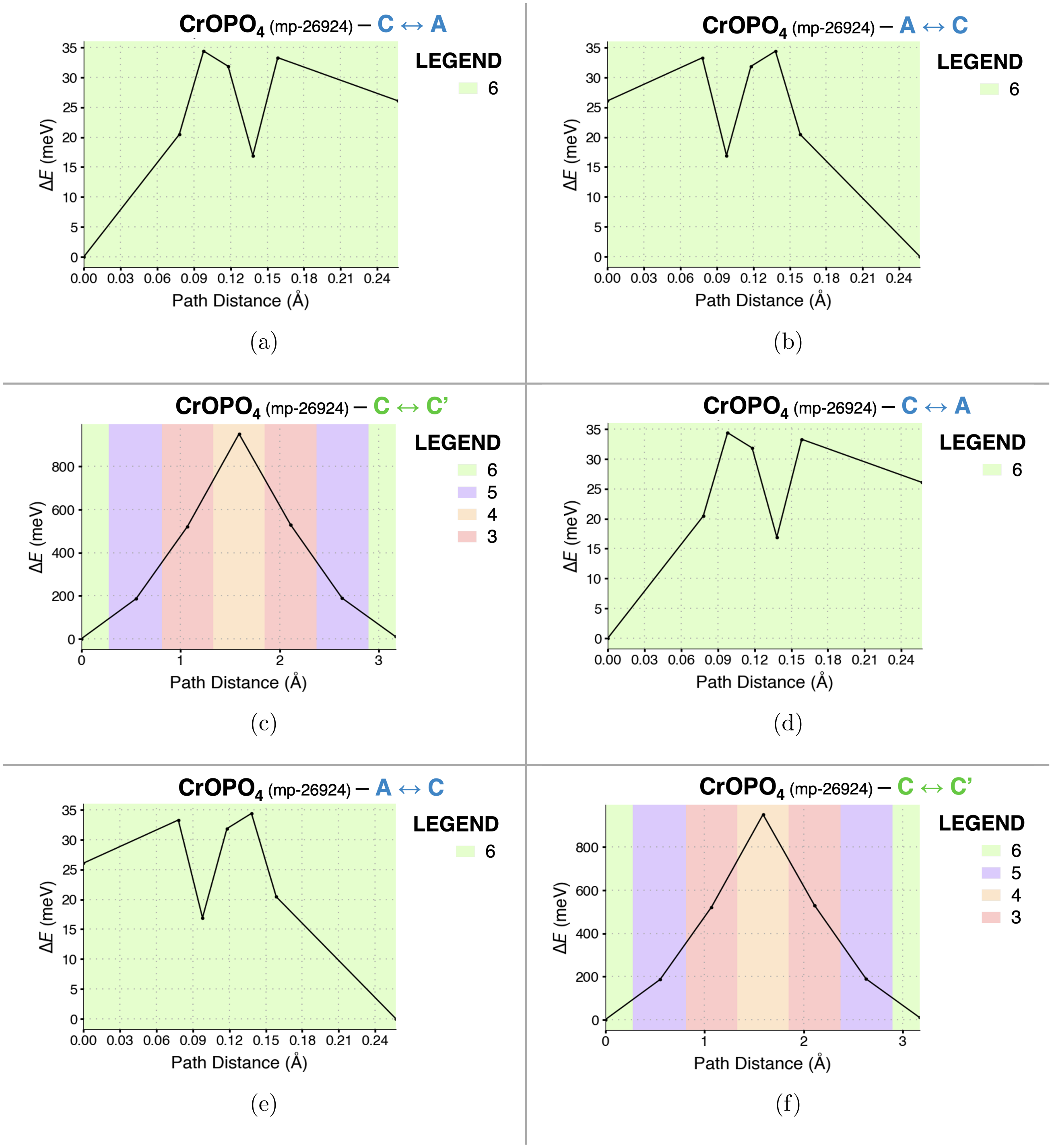

Figure S15: Energy landscape plots for Zn^{2+} migration along **PathwayII** in $CrOPO_4$ (mp-26924).

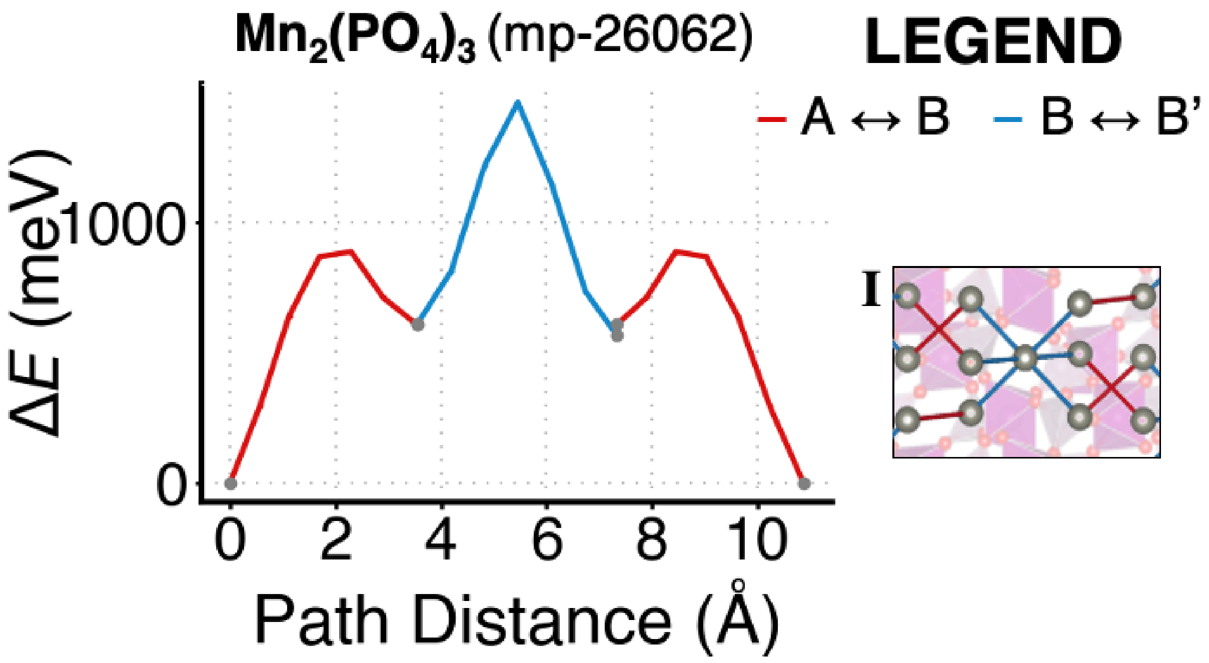

Figure S16: Energy landscape plots for trigonal NASICON $Mn_2(PO_4)_3$ (mp-26062), with an energetic barrier of 894 meV over a total path distance of 10.88 Å.

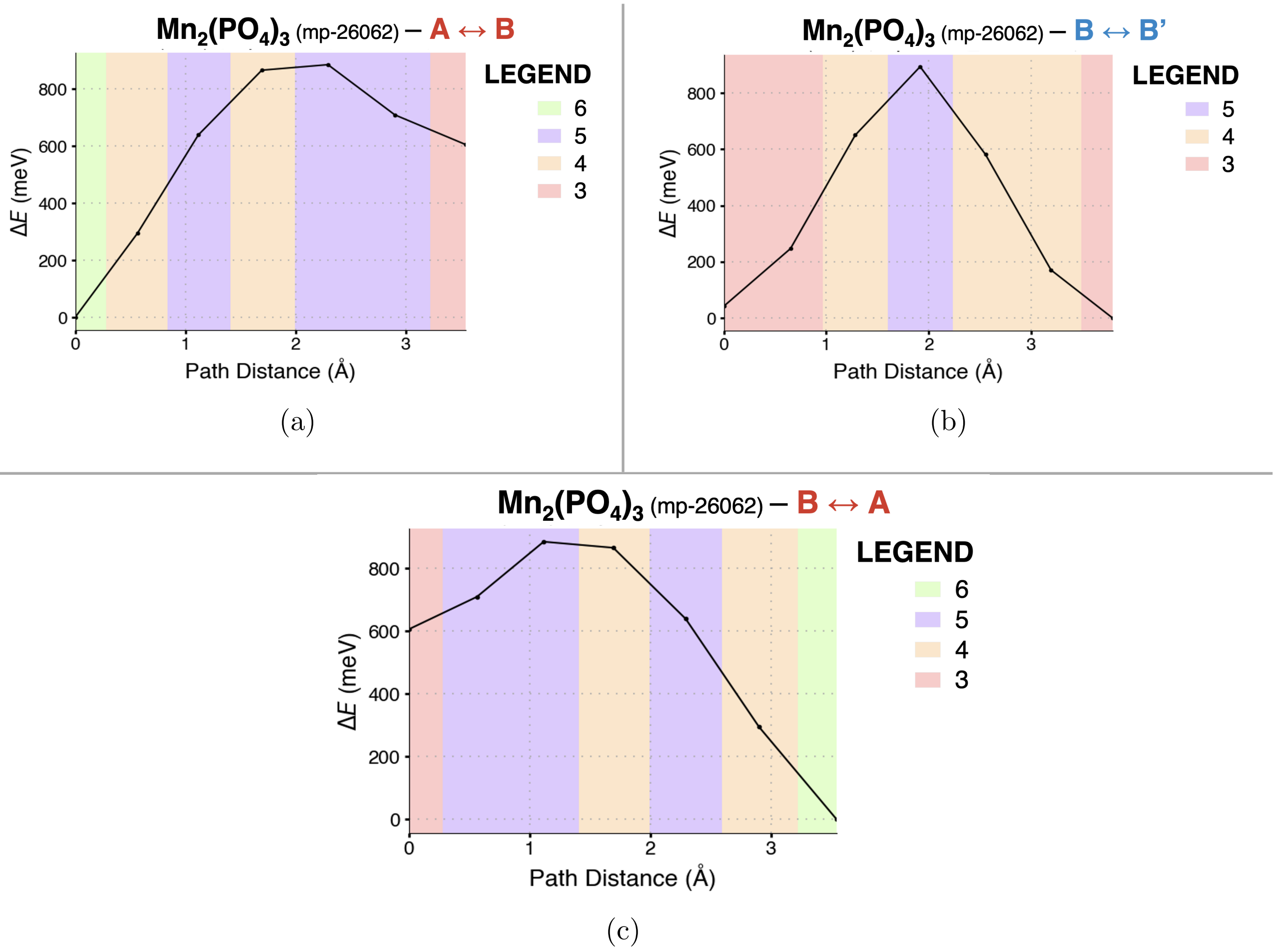

Figure S17: Energy landscape plots for Zn^{2+} migration along **PathwayI** in $Mn_2(PO_4)_3$ (mp-26062).

S4 Methodology/Computational Details

All electronic structure calculations were performed using the Perdew-Burke-Ernzerhof (PBE) generalized gradient approximation (GGA)[S50] in the Vienna *ab initio* simulation package (VASP)[S51–S54] version 6.3.2. For the insertion electrode calculations, accurate comparable energies across chemical systems were obtained by employing PBE in the absence of transition metal oxides and sulfides, PBE+U otherwise, and mixing energies from the two calculation methodologies using the established procedure in Materials Project.[S55,S56] All ApproxNEB calculations employed PBE without a Hubbard U correction. Although electronic self-interaction error tends to be highest in stretched radical bonds typical of a transition state,[S57] the optimal Hubbard U value to eliminate this self-interaction error cannot be calculated easily in high throughput and would require, e.g., a costly calculation via linear response.[S58] More, using a fixed value of U which is not optimized along the migration path has been shown not to be more performant in predicting accurate barriers via NEB and ApproxNEB.[S30,S59–S62] Chosen numerical and convergence parameters for these calculations are identical to those in previously published work,[S15,S63] employing the `MPRelaxSet` as implemented in `pymatgen`.[S5] The critical electronic self-consistency (`INCAR`) settings are summarized as follows: structural relaxation was performed until the total energy was converged within 5×10^{-4} eV and the forces on each atom were converged within 0.05 eV/Å; the plane-wave energy cutoff was set to 520 eV; all calculations used Gaussian smearing of the Fermi surface with smearing width 0.05 eV. The k-point density was set to be 64 points·Å^3, using the "reciprocal_density" tag in `pymatgen`. Consistent with Materials Project calculations and previous ApproxNEB calculations, we used the original PBE pseudopotentials (POTCARs) with release snapshot "06-05-2010". The ApproxNEB workflow implementation in the `atomate` workflow orchestration package[S64] is described in Ref. S14.

# Present addresses

§ Qian Chen: Toyota Research Institute of North America, Ann Arbor, Michigan 48105, United States

References

(S1) for Occupational Safety, T. N. I.; (NIOSH), H. Immediately Dangerous To Life or Health (IDLH) Values. https://archive.cdc.gov/www_cdc_gov/niosh/idlh/idlhintr.html, 2025.

(S2) Ong, S. P.; Wang, L.; Kang, B.; Ceder, G. Li- Fe- P- O2 phase diagram from first principles calculations. *Chemistry of Materials* **2008**, *20*, 1798–1807.

(S3) Hautier, G.; Ong, S. P.; Jain, A.; Moore, C. J.; Ceder, G. Accuracy of density functional theory in predicting formation energies of ternary oxides from binary oxides and its implication on phase stability. *Phys. Rev. B* **2012**, *85*, 155208.

(S4) Sun, W.; Dacek, S. T.; Ong, S. P.; Hautier, G.; Jain, A.; Richards, W. D.; Gamst, A. C.; Persson, K. A.; Ceder, G. The thermodynamic scale of inorganic crystalline metastability. *Science Advances* **2016**, *2*, e1600225.

(S5) Ong, S. P.; Richards, W. D.; Jain, A.; Hautier, G.; Kocher, M.; Cholia, S.; Gunter, D.; Chevrier, V. L.; Persson, K. A.; Ceder, G. Python Materials Genomics (pymatgen): A robust, open-source python library for materials analysis. *Computational Materials Science* **2013**, *68*, 314–319.

(S6) Persson, K. A.; Waldwick, B.; Lazic, P.; Ceder, G. Prediction of solid-aqueous equilibria: Scheme to combine first-principles calculations of solids with experimental aqueous states. *Physical Review B—Condensed Matter and Materials Physics* **2012**, *85*, 235438.

(S7) Singh, A. K.; Zhou, L.; Shinde, A.; Suram, S. K.; Montoya, J. H.; Winston, D.; Gregoire, J. M.; Persson, K. A. Electrochemical stability of metastable materials. *Chemistry of Materials* **2017**, *29*, 10159–10167.

(S8) Arthur, T. S.; Zhang, R.; Ling, C.; Glans, P.-A.; Fan, X.; Guo, J.; Mizuno, F. Understanding the Electrochemical Mechanism of K-αMnO2 for Magnesium Battery Cathodes. *ACS Applied Materials & Interfaces* **2014**, *6*, 7004–7008, PMID: 24807043.

(S9) Wang, X.; Xiao, R.; Li, H.; Chen, L. Quantitative structure-property relationship study of cathode volume changes in lithium ion batteries using ab-initio and partial least squares analysis. *Journal of Materiomics* **2017**, *3*, 178–183, High-throughput Experimental and Modeling Research toward Advanced Batteries.

(S10) Okada, S.; Sawa, S.; Egashira, M.; ichi Yamaki, J.; Tabuchi, M.; Kageyama, H.; Konishi, T.; Yoshino, A. Cathode properties of phospho-olivine LiMPO4 for lithium secondary batteries. *Journal of Power Sources* **2001**, *97-98*, 430–432, Proceedings of the 10th International Meeting on Lithium Batteries.

(S11) Ong, S. P.; Jain, A.; Hautier, G.; Kang, B.; Ceder, G. Thermal stabilities of delithiated olivine MPO4 (M=Fe, Mn) cathodes investigated using first principles calculations. *Electrochemistry Communications* **2010**, *12*, 427–430.

(S12) Hannah, D. C.; Sai Gautam, G.; Canepa, P.; Ceder, G. On the Balance of Intercalation and Conversion Reactions in Battery Cathodes. *Advanced Energy Materials* **2018**, *8*, 1800379.

(S13) Ming, J.; Guo, J.; Xia, C.; Wang, W.; Alshareef, H. N. Zinc-ion batteries: Materials, mechanisms, and applications. *Materials Science and Engineering: R: Reports* **2019**, *135*, 58–84.

(S14) Rutt, A.; Shen, J.-X.; Horton, M.; Kim, J.; Lin, J.; Persson, K. A. Expanding the

Material Search Space for Multivalent Cathodes. *ACS Applied Materials & Interfaces* **2022**, *14*, 44367–44376, PMID: 36137562.

(S15) Kim, J.; Sari, D.; Chen, Q.; Ceder, G.; Persson, K. A. Evaluating Material Design Principles for Calcium-Ion Mobility in Intercalation Cathodes. *Chemistry of Materials* **2025**, *37*, 507–519.

(S16) Masquelier, C.; Croguennec, L. Polyanionic (phosphates, silicates, sulfates) frameworks as electrode materials for rechargeable Li (or Na) batteries. *Chemical Reviews* **2013**, *113*, 6552–6591.

(S17) Shen, J.-X.; Horton, M.; Persson, K. A. A charge-density-based general cation insertion algorithm for generating new Li-ion cathode materials. *npj Computational Materials* **2020**, *6*, 161.

(S18) Fedotov, S. S.; Khasanova, N. R.; Samarin, A. S.; Drozhzhin, O. A.; Batuk, D.; Karakulina, O. M.; Hadermann, J.; Abakumov, A. M.; Antipov, E. V. AVPO4F (A= Li, K): a 4 V cathode material for high-power rechargeable batteries. *Chemistry of Materials* **2016**, *28*, 411–415.

(S19) Ahsan, Z.; Cai, Z.; Wang, S.; Moin, M.; Wang, H.; Liu, D.; Ma, Y.; Song, G.; Wen, C. Recent Development of Phosphate Based Polyanion Cathode Materials for Sodium-Ion Batteries. *Advanced Energy Materials* **2024**, *14*, 2400373.

(S20) Okada, S.; Ueno, M.; Uebou, Y.; Yamaki, J.-i. Fluoride phosphate Li2CoPO4F as a high-voltage cathode in Li-ion batteries. *Journal of power sources* **2005**, *146*, 565–569.

(S21) Schoiber, J.; Berger, R. J.; Bernardi, J.; Schubert, M.; Yada, C.; Miki, H.; Hüsing, N. Straightforward Solvothermal Synthesis toward Phase Pure Li2CoPO4F. *Crystal Growth & Design* **2016**, *16*, 4999–5005.

(S22) Hadermann, J.; Abakumov, A. M.; Turner, S.; Hafideddine, Z.; Khasanova, N. R.; Antipov, E. V.; Van Tendeloo, G. Solving the structure of Li ion battery materials with precession electron diffraction: Application to Li2CoPO4F. *Chemistry of Materials* **2011**, *23*, 3540–3545.

(S23) Pérez-Vicente, C.; Alcántara, R. New perspectives on the multianion approach to adapt electrode materials for lithium and post-lithium batteries. *Physical Chemistry Chemical Physics* **2023**, *25*, 15600–15623.

(S24) Fedotov, S. S.; Kabanov, A. A.; Kabanova, N. A.; Blatov, V. A.; Zhugayevych, A.; Abakumov, A. M.; Khasanova, N. R.; Antipov, E. V. Crystal structure and Li-ion transport in Li2CoPO4F high-voltage cathode material for Li-ion batteries. *The Journal of Physical Chemistry C* **2017**, *121*, 3194–3202.

(S25) Kubota, K.; Yokoh, K.; Yabuuchi, N.; Komaba, S. Na2CoPO4F as a high-voltage electrode material for Na-ion batteries. *Electrochemistry* **2014**, *82*, 909–911.

(S26) Zou, H.; Li, S.; Wu, X.; McDonald, M. J.; Yang, Y. Spray-drying synthesis of pure Na2CoPO4F as cathode material for sodium ion batteries. *ECS Electrochemistry Letters* **2015**, *4*, A53.

(S27) Chernova, N. A.; Hidalgo, M. F. V.; Kaplan, C.; Lee, K.; Buyuker, I.; Siu, C.; Wen, B.; Ding, J.; Zuba, M.; Wiaderek, K. M.; others Vanadyl phosphates AxVOPO4 (A= Li, Na, K) as multielectron cathodes for alkali-ion batteries. *Advanced Energy Materials* **2020**, *10*, 2002638.

(S28) Ma, S.; Jiang, T.; Deng, J.; Zhang, Q.; Ou, Y.; Liu, X.; Lin, C.; Wang, K.; Zhao, X. VPO5: an all-climate lithium-storage material. *Energy Storage Materials* **2022**, *46*, 366–373.

(S29) Shen, J.-X.; Li, H. H.; Rutt, A.; Horton, M. K.; Persson, K. A. Topological graph-based analysis of solid-state ion migration. *npj Computational Materials* **2023**, *9*, 99.

(S30) Sari, D.; Rutt, A.; Kim, J.; Chen, Q.; Hahn, N. T.; Kim, H.; Persson, K. A.; Ceder, G. Alkali-Ion-Assisted Activation of ε-VOPO4 as a Cathode Material for Mg-Ion Batteries. *Advanced Science* **2024**, 2307838.

(S31) Zhao, D.; Pu, X.; Tang, S.; Ding, M.; Zeng, Y.; Cao, Y.; Chen, Z. δ-VOPO 4 as a high-voltage cathode material for aqueous zinc-ion batteries. *Chemical Science* **2023**, *14*, 8206–8213.

(S32) Kaplan, C.; Hidalgo, M. F. V.; Zuba, M. J.; Chernova, N. A.; Piper, L. F.; Whittingham, M. S. Microwave-assisted solvothermal synthesis of LiV y M 1- y OPO 4 (M= Mn, Cr, Ti, Zr, Nb, Mo, W) cathode materials for lithium-ion batteries. *Journal of Materials Chemistry A* **2021**, *9*, 6933–6944.

(S33) Mueller, T.; Hautier, G.; Jain, A.; Ceder, G. Evaluation of tavorite-structured cathode materials for lithium-ion batteries using high-throughput computing. *Chemistry of materials* **2011**, *23*, 3854–3862.

(S34) Lu, M. Y.; Badway, F.; Kim, J. R.; Amatucci, G. G. Investigation of Physical and Electrochemical Properties of β-Ta x Nb1–x PO5 as an Electrode Material for Lithium Batteries. *Chemistry of materials* **2016**, *28*, 2949–2961.

(S35) Aparicio, P. A.; Dawson, J. A.; Islam, M. S.; De Leeuw, N. H. Computational study of NaVOPO4 polymorphs as cathode materials for Na-ion batteries: Diffusion, electronic properties, and cation-doping behavior. *The Journal of Physical Chemistry C* **2018**, *122*, 25829–25836.

(S36) Ren, M.; Zhou, Z.; Su, L.; Gao, X. LiVOPO4: A cathode material for 4 V lithium ion batteries. *Journal of Power Sources* **2009**, *189*, 786–789.

(S37) Hautier, G.; Jain, A.; Ong, S. P.; Kang, B.; Moore, C.; Doe, R.; Ceder, G. Phosphates as lithium-ion battery cathodes: an evaluation based on high-throughput ab initio calculations. *Chemistry of Materials* **2011**, *23*, 3495–3508.

(S38) Wang, J.; He, T.; Yang, X.; Cai, Z.; Wang, Y.; Lacivita, V.; Kim, H.; Ouyang, B.; Ceder, G. Design principles for NASICON super-ionic conductors. *Nature Communications* **2023**, *14*, 5210.

(S39) Liu, Y.; Li, J.; Shen, Q.; Zhang, J.; He, P.; Qu, X.; Liu, Y. Advanced characterizations and measurements for sodium-ion batteries with NASICON-type cathode materials. *EScience* **2022**, *2*, 10–31.

(S40) Zhou, Y.; Xu, G.; Lin, J.; Zhang, Y.; Fang, G.; Zhou, J.; Cao, X.; Liang, S. Reversible Multielectron Redox Chemistry in a NASICON-Type Cathode toward High-Energy-Density and Long-Life Sodium-Ion Full Batteries. *Advanced Materials* **2023**, *35*, 2304428.

(S41) Masquelier, C.; Padhi, A.; Nanjundaswamy, K.; Goodenough, J. New cathode materials for rechargeable lithium batteries: the 3-D framework structures Li3Fe2 (XO4) 3 (X= P, As). *Journal of Solid State Chemistry* **1998**, *135*, 228–234.

(S42) Thirupathi, R.; Kumari, V.; Chakrabarty, S.; Omar, S. Recent progress and prospects of NASICON framework electrodes for Na-ion batteries. *Progress in Materials Science* **2023**, *137*, 101128.

(S43) Zhao, X.; Zhang, Z.; Zhang, X.; Tang, B.; Xie, Z.; Zhou, Z. Computational screening and first-principles investigations of NASICON-type Li x M 2 (PO 4) 3 as solid electrolytes for Li batteries. *Journal of Materials Chemistry A* **2018**, *6*, 2625–2631.

(S44) Wu, M.; Ni, W.; Hu, J.; Ma, J. NASICON-structured NaTi 2 (PO 4) 3 for sustainable energy storage. *Nano-Micro Letters* **2019**, *11*, 1–36.

(S45) Gao, H.; Li, Y.; Park, K.; Goodenough, J. B. Sodium extraction from NASICON-structured Na3MnTi (PO4) 3 through Mn (III)/Mn (II) and Mn (IV)/Mn (III) redox couples. *Chemistry of Materials* **2016**, *28*, 6553–6559.

(S46) Li, H.; Jin, T.; Chen, X.; Lai, Y.; Zhang, Z.; Bao, W.; Jiao, L. Rational architecture design enables superior Na storage in greener NASICON-Na4MnV (PO4) 3 cathode. *Advanced Energy Materials* **2018**, *8*, 1801418.

(S47) Zhou, W.; Xue, L.; Lü, X.; Gao, H.; Li, Y.; Xin, S.; Fu, G.; Cui, Z.; Zhu, Y.; Goodenough, J. B. Na x MV (PO4) 3 (M= Mn, Fe, Ni) structure and properties for sodium extraction. *Nano letters* **2016**, *16*, 7836–7841.

(S48) Wu, B.; Hou, G.; Kovalska, E.; Mazanek, V.; Marvan, P.; Liao, L.; Dekanovsky, L.; Sedmidubsky, D.; Marek, I.; Hervoches, C.; others High-Entropy NASICON Phosphates (Na3M2 (PO4) 3 and NaMPO4O x, M= Ti, V, Mn, Cr, and Zr) for Sodium Electrochemistry. *Inorganic Chemistry* **2022**, *61*, 4092–4101.

(S49) Patra, B.; Kumar, K.; Deb, D.; Ghosh, S.; Gautam, G. S.; Senguttuvan, P. Unveiling a high capacity multi-redox (Nb 5+/Nb 4+/Nb 3+) NASICON-Nb 2 (PO 4) 3 anode for Li-and Na-ion batteries. *Journal of Materials Chemistry A* **2023**, *11*, 8173–8183.

(S50) Perdew, J. P.; Burke, K.; Ernzerhof, M. Generalized gradient approximation made simple. *Phys. Rev. Lett.* **1996**, *77*, 3865.

(S51) Kresse, G.; Hafner, J. *Ab initio* molecular dynamics for liquid metals. *Physical Review B* **1993**, *47*, 558–561.

(S52) Kresse, G.; Hafner, J. *Ab initio* molecular-dynamics simulation of the liquid-metal–amorphous-semiconductor transition in germanium. *Physical Review B* **1994**, *49*, 14251–14269.

(S53) Kresse, G.; Furthmüller, J. Efficient iterative schemes for *ab initio* total-energy calculations using a plane-wave basis set. *Physical Review B* **1996**, *54*, 11169–11186.

(S54) Kresse, G.; Furthmüller, J. Efficiency of ab-initio total energy calculations for metals

and semiconductors using a plane-wave basis set. *Computational Materials Science* **1996**, *6*, 15–50.

(S55) Jain, A.; Ong, S. P.; Hautier, G.; Chen, W.; Richards, W. D.; Dacek, S.; Cholia, S.; Gunter, D.; Skinner, D.; Ceder, G.; others Commentary: The Materials Project: A materials genome approach to accelerating materials innovation. *APL materials* **2013**, *1*, 011002.

(S56) Jain, A.; Hautier, G.; Ong, S. P.; Moore, C. J.; Fischer, C. C.; Persson, K. A.; Ceder, G. Formation enthalpies by mixing GGA and GGA + U calculations. *Phys. Rev. B* **2011**, *84*, 045115.

(S57) Kaplan, A. D.; Shahi, C.; Bhetwal, P.; Sah, R. K.; Perdew, J. P. Understanding Density-Driven Errors for Reaction Barrier Heights. *J. Chem. Theory Comput.* **2023**, *19*, 532–543.

(S58) Cococcioni, M.; de Gironcoli, S. Linear response approach to the calculation of the effective interaction parameters in the LDA + U method. *Phys. Rev. B* **2005**, *71*, 035105.

(S59) Morgan, D. AV d. Ven and G. Ceder. *Electrochem. Solid-State Lett* **2004**, *7*, A30–A32.

(S60) Liu, M.; Rong, Z.; Malik, R.; Canepa, P.; Jain, A.; Ceder, G.; Persson, K. A. Spinel compounds as multivalent battery cathodes: a systematic evaluation based on ab initio calculations. *Energy & Environmental Science* **2015**, *8*, 964–974.

(S61) Dathar, G. K. P.; Sheppard, D.; Stevenson, K. J.; Henkelman, G. Calculations of Li-ion diffusion in olivine phosphates. *Chemistry of Materials* **2011**, *23*, 4032–4037.

(S62) Ong, S. P.; Chevrier, V. L.; Hautier, G.; Jain, A.; Moore, C.; Kim, S.; Ma, X.; Ceder, G. Voltage, stability and diffusion barrier differences between sodium-ion and

lithium-ion intercalation materials. *Energy & Environmental Science* **2011**, *4*, 3680–3688.

(S63) Kim, J.; Sari, D.; Chen, Q.; Rutt, A.; Ceder, G.; Persson, K. A. First-Principles and Experimental Investigation of ABO4 Zircons as Calcium Intercalation Cathodes. *Chemistry of Materials* **2024**, *36*, 4444–4455.

(S64) Mathew, K.; Montoya, J. H.; Faghaninia, A.; Dwarakanath, S.; Aykol, M.; Tang, H.; heng Chu, I.; Smidt, T.; Bocklund, B.; Horton, M.; Dagdelen, J.; Wood, B.; Liu, Z.-K.; Neaton, J.; Ong, S. P.; Persson, K.; Jain, A. Atomate: A high-level interface to generate, execute, and analyze computational materials science workflows. *Computational Materials Science* **2017**, *139*, 140–152.
